# Posterior Circulation Aneurysmal Subarachnoid Hemorrhage: A Comparison Between Saccular and Dissecting Aneurysms

**DOI:** 10.1002/brb3.71668

**Published:** 2026-09-07

**Authors:** Jian Li, Guoqiang Song, Guijing Liu, Yunyi Tong, Xinmiao Gu, Bangyue Wang, Libin Fan, Letian Li, Xinyu Yang, Honglei Liang, Xiaojun Zhang

**Affiliations:** ^1^ Department of Neurosurgery Tianjin Medical University General Hospital Tianjin China; ^2^ Department of Neurosurgery The Second Hospital of Hebei Medical University Shijiazhuang China; ^3^ Kunshan Traditional Chinese Medicine Hospital Suzhou Jiangsu Province China; ^4^ Department of Neurology Tianjin Medical University General Hospital Tianjin China; ^5^ Department of Neurosurgery The Second Affiliated Hospital of Anhui Medical University Hefei China; ^6^ Department of Neurosurgery The People's Hospital of Anyang City Anyang China; ^7^ Department of Neurosurgery Tongji University Affiliated Tianyou Hospital Shanghai China

**Keywords:** aneurysmal subarachnoid hemorrhage, dissecting aneurysm, outcome, posterior circulation aneurysm, saccular aneurysm

## Abstract

**Background:**

There is a lack of comparative studies between posterior circulation saccular aneurysms (SA) and dissecting aneurysms (DA). This study aims to comprehensively compare the clinical characteristics, treatment strategies, and outcomes of SA and DA.

**Methods:**

We included all consecutive patients with posterior circulation aneurysmal subarachnoid hemorrhage (aSAH) who underwent surgical treatment between January 2017 and December 2020 from the Chinese Multicenter Cerebral Aneurysm Database (CMAD). Baseline data were retrospectively collected, and survival status and 2‐year mRS scores were prospectively assessed. Functional outcomes were categorized as favorable (mRS 0–2) and unfavorable (mRS 3–6). Logistic regression models were used to explore the association between aneurysm morphology and outcomes.

**Results:**

Note that 406 patients with posterior circulation aSAH who underwent surgical treatment were included, comprising 314 (77.3%) with SA and 92 (22.7%) with DA. In unadjusted analyses, DA was associated with a lower rate of unfavorable outcomes at 2 years (17.1% vs. 34.8%, *p* = 0.003) but a higher rate of parent vessel sacrifice (22.8% vs. 8.0%, *p* < 0.001), while ischemic complications were comparable between groups. After further adjustment for covariates, DA showed a trend toward a lower risk of unfavorable outcomes in Model 3 (OR = 0.490, 95% CI 0.237–1.013, *p* = 0.054). In addition, the association between DA and a higher risk of parent vessel sacrifice remained consistent across all models (Model 1: OR = 3.419, 95% CI 1.811–6.456, *p* < 0.001; Model 2: OR = 2.737, 95% CI 1.415–5.293, *p* = 0.003; Model 3: OR = 2.899, 95% CI 1.463–5.747, *p* = 0.002).

**Conclusion:**

DA may be associated with a trend toward better long‐term functional outcomes compared with SA. Although parent vessel sacrifice was more frequently required, it was not associated with an increased risk of ischemic complications.

## Graphical abstract

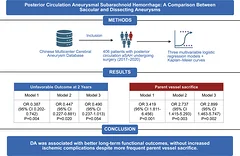

## 1 Introduction

Aneurysmal subarachnoid hemorrhage (aSAH) is a devastating neurological condition associated with high morbidity and mortality (de Rooij et al. 2007; Nieuwkamp et al. 2009). Posterior circulation aneurysms account for approximately 10% of all intracranial aneurysms (Rinkel et al. 1998). Due to the complex anatomy and limited space of the posterior fossa, as well as its proximity to the brainstem and the abundance of critical perforating arteries, the treatment of posterior circulation aSAH is technically challenging and carries a higher procedural risk, often resulting in poorer overall outcomes (Liang et al. 2019; Schievink et al. 1995; Dharia et al. 2020; Abla et al. 2014).

Posterior circulation aneurysms can be morphologically classified into saccular aneurysms (SA) and dissecting aneurysms (DA) (Maimon et al. 2006). These two types differ substantially in terms of pathogenesis, vascular wall alterations, rupture patterns, and the risk of rebleeding (Krings and Choi 2010; Mizutani 2011; Park et al. 2008; Sikkema et al. 2014; Santos‐Franco et al. 2008). DA are relatively rare, accounting for approximately 3% of all intracranial aneurysms (Santos‐Franco et al. 2008). However, they are disproportionately represented in the posterior circulation, with previous studies reporting that 76%–93% of intracranial DA occur in this vascular territory (Mizutani 2011). Despite several studies investigating posterior circulation aSAH (Baker et al. 2020; Steiger et al. 1999; Tykocki and Kostkiewicz 2014), the rarity of these cases and the limited number of direct comparisons between SA and DA have resulted in insufficient evidence regarding optimal treatment strategies and long‐term outcomes. Based on multicenter data, the present study aimed to comprehensively compare the clinical characteristics, treatment strategies, and outcomes of SA and DA.

## 2 Methods

Patients with posterior circulation aSAH who underwent surgical treatment between January 2017 and December 2020 were identified from the Chinese Multicenter Cerebral Aneurysm Database (CMAD). CMAD is a registered, multicenter, observational database in China. The study design was approved by the Ethics Committee of Tianjin Medical University General Hospital (IRB2022‐YX‐175‐01). As this was an observational study using routinely collected clinical data, the requirement for informed consent was waived. All analyses were conducted in accordance with the Declaration of Helsinki and local ethical regulations.

### 2.1 Data Collection

Demographic data (age, sex, residence, smoking history, and alcohol use) and clinical data (hypertension, diabetes, prior stroke, presence of intracerebral hemorrhage [ICH] or intraventricular hemorrhage [IVH], Hunt–Hess [HH] grade, WFNS grade, location and morphology of the responsible aneurysm, number of aneurysms, treatment modality, in‐hospital mortality, discharge modified Rankin Scale (mRS) score, parent vessel sacrifice, tracheostomy, decompressive craniectomy, and ventriculoperitoneal shunt) were collected retrospectively. Between 2022 and 2025, survival status and 2‐year mRS were collected prospectively through standardized telephone follow‐up. The mRS is a 7‐point scale ranging from 0 (no symptoms) to 6 (death) and was assessed by a member of the study team. Functional outcomes were categorized as favorable (mRS 0–2) and unfavorable (mRS 3–6).

### 2.2 Statistical Analysis

All statistical analyses were performed using SPSS version 27 (IBM Corp, Armonk, NY, USA) and R version 4.4.1 (R Foundation for Statistical Computing, Vienna, Austria). For 95% confidence intervals (CI), two‐sided *p*‐values < 0.05 were considered statistically significant.

Continuous variables were presented as mean ± standard deviation (SD), and categorical variables as counts (percentages). Comparisons of categorical variables were performed using the Pearson chi‐square test or Fisher's exact test, as appropriate. Survival between the SA and DA groups was compared using Kaplan–Meier curves.

Multivariable logistic regression models were used to examine the association between aneurysm morphology and outcomes. Three models were constructed: Model 1 was unadjusted; Model 2 was adjusted for age, sex, and hypertension; and Model 3 was further adjusted for HH grade and treatment modality based on Model 2. Firth penalized logistic regression was applied for outcomes with zero events in the DA group, including rebleeding, decompressive craniectomy, and ventriculoperitoneal shunt placement.

Sensitivity analyses were performed to evaluate the potential impact of loss to follow‐up on the robustness of the primary findings regarding 2‐year functional outcomes. Two extreme scenarios were applied: (1) a best‐case scenario, assuming that all patients lost to follow‐up had favorable outcomes; and (2) a worst‐case scenario, assuming that all patients lost to follow‐up had unfavorable outcomes. Logistic regression models were constructed under each scenario to assess the association between aneurysm morphology and 2‐year unfavorable outcomes, with adjustment for age, sex, hypertension, HH grade, and treatment modality.

## 3 Results

A total of 406 patients with posterior circulation aSAH who underwent surgical treatment were included, comprising 314 (77.3%) with SA and 92 (22.7%) with DA. Compared with the SA group, patients in the DA group were younger, had a lower proportion of basilar artery (BA) aneurysms, and underwent microsurgical treatment (MST) less frequently, whereas the proportion of males and vertebral artery (VA) aneurysms was higher (Table 1).

**TABLE 1:** Comparison of baseline characteristics between the SA and DA groups.

	Total (*n* = 406)	SA (*n* = 314)	DA (*n* = 92)	*p*‐value
Age, y; (mean, SD)	58.9 (11.9)	60.5 (11.4)	53.7 (12.2)	
< 65	255 (62.8)	184 (58.6)	71 (77.2)	0.001
≥ 65	151 (37.2)	130 (41.4)	21 (22.8)	
Sex, *n* (%)				0.007
Male	163 (40.1)	115 (36.6)	48 (52.2)	
Female	243 (59.9)	199 (63.4)	44 (47.8)	
Smoking, *n* (%)	69 (17.0)	49 (15.6)	20 (21.7)	0.168
Drinking, *n* (%)	48 (11.8)	33 (10.5)	15 (16.3)	0.130
Hypertension, *n* (%)	264 (65.0)	214 (68.2)	50 (54.3)	0.015
Diabetes, *n* (%)	46 (11.3)	38 (12.1)	8 (8.7)	0.365
Previous stroke, *n* (%)	59 (14.5)	45 (14.3)	14 (15.2)	0.832
Presence of ICH or IVH, *n* (%)	100 (24.6)	74 (23.6)	26 (28.3)	0.358
HH grade, *n* (%)				0.180
I–III	334 (82.3)	254 (80.9)	80 (87.0)	
IV–V	72 (17.7)	60 (19.1)	12 (13.0)	
WFNS grade, *n* (%)				0.078
I–III	288 (70.9)	216 (68.8)	72 (78.3)	
IV–V	118 (29.1)	98 (31.2)	20 (21.7)	
Location, *n* (%)				< 0.001
BA	131 (32.3)	121 (38.5)	10 (10.9)	
SCA	16 (3.9)	15 (4.8)	1 (1.1)	
PICA	88 (21.7)	80 (25.5)	8 (8.7)	
VA	86 (21.2)	28 (8.9)	58 (63.0)	
Vertebrobasilar junction	6 (1.5)	5 (1.6)	1 (1.1)	
PCA	68 (16.7)	57 (18.2)	11 (12.0)	
AICA	11 (2.7)	8 (2.5)	3 (3.3)	
Multiple aneurysms, *n* (%)	90 (22.2)	72 (22.9)	18 (19.6)	0.494
Treatment, *n* (%)				< 0.001
MST	34 (8.4)	34 (10.8)	0	
EVT	372 (91.6)	280 (89.2)	92 (100.0)	

Abbreviations: AICA, anterior inferior cerebellar artery; BA, basilar artery; DA, dissecting aneurysm; EVT, endovascular treatment; HH, Hunt‐Hess; ICH, intracerebral hemorrhage; IVH, intraventricular hemorrhage; MST, microsurgical treatment; PCA, posterior cerebral artery; PICA; posterior inferior cerebellar artery; SA, saccular aneurysm; SCA, superior cerebellar artery; VA, vertebral artery.

**FIGURE 1:**
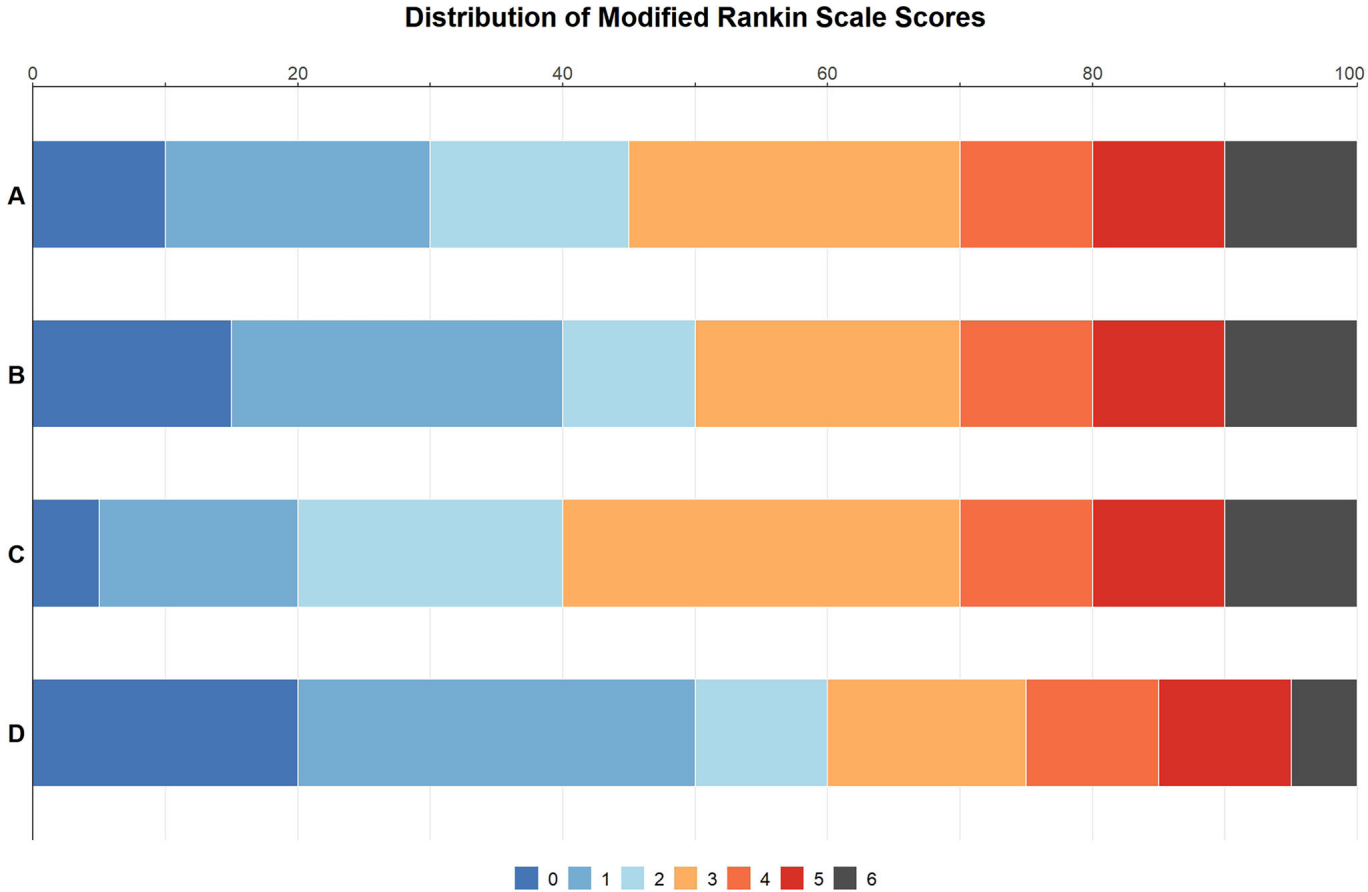
The distribution of mRS scores in the SA and DA groups. (A) SA group at discharge; (B) DA group at discharge; (C) SA group at 2 years; (D) DA group at 2 years.

No significant differences were observed between the SA and DA groups in functional outcomes at discharge, in‐hospital mortality, rebleeding, or ischemic complications. A total of 332 patients completed the 2‐year follow‐up, of whom 102 (30.7%) had unfavorable outcomes and 62 (18.7%) had died. Baseline characteristics did not differ significantly between patients who completed follow‐up and those lost to follow‐up (Table S1). Compared with the SA group, the DA group had a lower incidence of unfavorable outcomes at 2 years (17.1% vs. 34.8%, *p* = 0.003) and lower 2‐year mortality (10.5% vs. 21.1%, *p* = 0.038) (Table 2). The distribution of mRS scores in the SA and DA groups is shown in Figure 1.

**TABLE 2:** Distribution of outcomes between the SA and DA groups.

	Total	SA	DA	*p*‐value
Discharge				
No. of patients	406	314	92	
Function outcome, *n* (%)				0.374
Favorable outcome	308 (75.9)	235 (74.8)	73 (79.3)	
Unfavorable outcome	98 (24.1)	79 (25.2)	19 (20.7)	
Survival outcomes, *n* (%)				1
Survival	393 (96.8)	304 (96.8)	89 (96.7)	
Mortality	13 (3.2)	10 (3.2)	3 (3.3)	
In‐hospital complications, *n* (%)				
Rebleeding	6 (1.5)	6 (1.9)	0	0.398
Cerebral ischemia	51 (12.6)	40 (12.7)	11 (12.0)	0.842
2 year				
No. of patients	332	256	76	
Function outcome, *n* (%)				0.003
Favorable outcome	230 (69.3)	167 (65.2)	63 (82.9)	
Unfavorable outcome	102 (30.7)	89 (34.8)	13 (17.1)	
Survival outcomes, *n* (%)				0.038
Survival	270 (81.3)	202 (78.9)	68 (89.5)	
Mortality	62 (18.7)	54 (21.1)	8 (10.5)	

Among patients undergoing surgery for posterior circulation aSAH, parent vessel sacrifice was performed in 46 cases (11.3%). The proportion of parent vessel sacrifice was higher in the DA group compared with the SA group (22.8% vs. 8.0%, *p* < 0.001) (Table 3).

**TABLE 3:** Comparison of clinical characteristics and treatment‐related procedures between SA and DA.

	Total (*n* = 406)	SA (*n* = 314)	DA (*n* = 92)	*p*‐value
Parent vessel sacrifice	46 (11.3)	25 (8.0)	21 (22.8)	< 0.001
Tracheostomy	13 (3.2)	12 (3.8)	1 (1.1)	0.330
Decompressive craniectomy	6 (1.5)	6 (1.9)	0	0.398
Ventriculoperitoneal shunt	7 (1.7)	7 (2.2)	0	0.323

**FIGURE 2:**
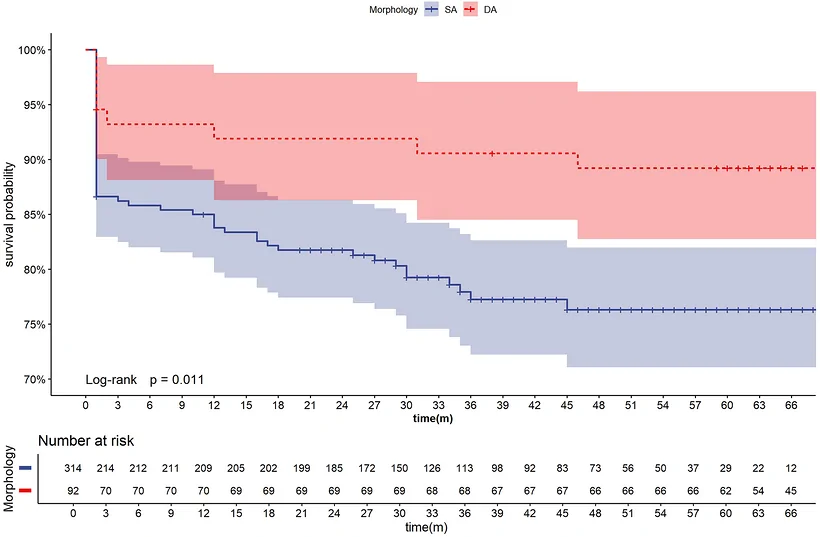
Kaplan–Meier survival curves comparing long‐term survival between saccular and dissecting posterior circulation aneurysms.

Kaplan–Meier survival analysis included all 406 patients, with a mean follow‐up of 34.05 ± 28.41 months. The 2‐year cumulative mortality was significantly higher in the SA group compared with the DA group (*p* = 0.011) (Figure 2).

In the regression analysis, Model 1 showed that DA was significantly associated with a lower risk of 2‐year unfavorable outcomes (OR = 0.387, 95% CI 0.202–0.742, *p* = 0.004) and 2‐year mortality (OR = 0.440, 95% CI 0.199–0.971, *p* = 0.042). However, after further adjustment for covariates in Models 2 and 3, the association between DA and 2‐year mortality was no longer statistically significant (Model 2: OR = 0.541, 95% CI 0.236–1.243, *p* = 0.148; Model 3: OR = 0.631, 95% CI 0.266–1.496, *p* = 0.296). Regarding unfavorable outcomes, the association between DA and better functional outcomes remained significant in Model 2 (OR = 0.447, 95% CI 0.227–0.881, *p* = 0.020) but was attenuated and showed a trend toward significance after further adjustment in Model 3 (OR = 0.490, 95% CI 0.237–1.013, *p* = 0.054). In addition, DA was consistently associated with a higher risk of parent vessel sacrifice across all models (Model 1: OR = 3.419, 95% CI 1.811–6.456, *p* < 0.001; Model 2: OR = 2.737, 95% CI 1.415–5.293, *p* = 0.003; Model 3: OR = 2.899, 95% CI 1.463–4.747, *p* = 0.002) (Table 4). In the sensitivity analyses, the association between DA and unfavorable outcomes remained directionally consistent but did not reach statistical significance under either the best‐case or worst‐case assumptions (*p* = 0.155 and *p* = 0.063, respectively) (Figure S1).

**TABLE 4:** Association between aneurysm morphology and outcome.

	Model 1		Model 2		Model 3	
	OR (95% CI)	*p*‐value	OR (95% CI)	*p*‐value	OR (95% CI)	*p*‐value
SA	Reference		Reference		Reference	
Discharge						
Unfavorable outcome	0.774 (0.440–1.363)	0.375	0.858 (0.4781.539)	0.607	1.036 (0.536–2.001)	0.916
Mortality	1.025 (0.276–3.804)	0.971	1.295 (0.331–5.073)	0.710	1.700 (0.397–7.284)	0.475
Rebleeding^a^	0.257 (0.002–2.203)	0.262	0.227 (0.002–2.051)	0.225	0.199 (0.002–1.795)	0.179
Cerebral ischemia	0.930 (0.457–1.896)	0.842	1.015 (0.490–2.103)	0.968	1.049 (0.499–2.201)	0.900
2 year						
Unfavorable outcome	0.387 (0.202–0.742)	0.004	0.447 (0.227–0.881)	0.020	0.490 (0.237–1.013)	0.054
Mortality	0.440 (0.199–0.971)	0.042	0.541 (0.236–1.243)	0.148	0.631 (0.266–1.496)	0.296
Treatment‐related procedures						
Parent vessel sacrifice	3.419 (1.811–6.456)	< 0.001	2.737 (1.415–5.293)	0.003	2.899 (1.463–5.747)	0.002
Tracheostomy	0.277 (0.035–2.156)	0.220	0.340 (0.043–2.690)	0.307	0.479 (0.057–4.036)	0.499
Decompressive craniectomy^a^	0.257 (0.002–2.203)	0.262	0.248 (0.002–2.241)	0.259	1.057 (0.007–21.578)	0.974
Ventriculoperitoneal shunt^a^	0.222 (0.002–1.848)	0.200	0.335 (0.003–3.030)	0.394	0.307 (0.002–2.865)	0.357

*Note*: Model 1 was unadjusted. Model 2 was adjusted for age, sex, and hypertension. Model 3 was further adjusted for HH grade and treatment modality based on Model 2.

^a^ Firth penalized logistic regression was applied for rare event outcomes, including rebleeding, decompressive craniectomy, and ventriculoperitoneal shunt placement.

## 4 Discussion

Our study demonstrated that, among patients with posterior circulation aSAH, DA was associated with a trend toward better 2‐year functional outcomes compared with SA, although this association was attenuated after adjustment for potential confounders. DA was also associated with a higher likelihood of parent vessel sacrifice, whereas ischemic complications and 2‐year mortality were comparable between the two groups. In addition, the two groups differed in baseline characteristics, including age, sex, predominant aneurysm location, history of hypertension, and treatment modality. To our knowledge, this is the first study to compare clinical outcomes between posterior circulation SA and DA, providing additional evidence to inform clinical decision‐making.

In our cohort, DA and SA showed distinct anatomical distribution patterns. DA predominantly involved the VA, whereas SA was more frequently located in the BA. This finding is consistent with previous reports indicating that VA DAs represent the majority of posterior circulation DAs, while BA DAs are relatively uncommon (Balik et al. 2018). We also observed differences in baseline characteristics between the two groups. Compared with SA, patients with DA were generally younger, more frequently male, and had a higher prevalence of hypertension, which is consistent with previous studies (Amin‐Hanjani et al. 1997; Jin et al. 2009). Although the etiology of DA remains unclear in most cases, hypertension may be a risk factor for VA DAs (Jaja et al. 2015). Patients with hypertension have higher rates of comorbidities, are more likely to present with poor clinical grades, and are at greater risk of neurological complications (Roberts 1975). Foster et al. (2018) also reported that patients with DA are more likely to have worse clinical grades. However, in the present study, no significant differences in clinical grading were observed between the SA and DA groups. Although our study included a larger sample than previous reports, further large‐scale prospective studies are needed to provide meaningful insights.

Posterior circulation location has been identified as an independent risk factor for poor outcomes after both MST and endovascular treatment (EVT) (Wiebers 2003). Maduri et al. (2019) reported that two‐thirds of patients with posterior circulation aSAH achieved favorable neurological outcomes at 1‐year follow‐up. Similarly, in our study, 69.3% of patients had favorable outcomes at 2 years. Some studies have indicated that non‐SA in the vertebrobasilar system has a poor prognosis, with a reported median survival of 7.8 years (Flemming et al. 2005). However, Foster et al. (2018) found that discharge outcomes and 30‐day mortality were comparable between SA and DA, which is consistent with our findings. Interestingly, DA patients appeared to have more favorable functional outcomes in unadjusted analyses. After adjustment, this association was attenuated and remained only as a trend. Sensitivity analyses yielded consistent directional findings but did not demonstrate statistically significant differences. Similarly, although unadjusted survival analyses suggested a more favorable survival profile in patients with DA, the difference in 2‐year mortality was no longer significant after adjustment. This indicates that the apparent survival advantage observed in the unadjusted analysis may be partly explained by differences in patient characteristics and treatment selection rather than an independent effect of aneurysm morphology. These findings are partially consistent with the observations of Qi et al. (2023), who reported favorable clinical outcomes and acceptable complication rates after flow diverter treatment for posterior circulation aneurysms, particularly among dissecting and non‐basilar aneurysms. Previous studies have suggested that the treatment feasibility of DA, including the potential use of parent vessel sacrifice and favorable collateral circulation in selected patients, may contribute to acceptable clinical outcomes (Griessenauer et al. 2019; Griessenauer et al. 2020). Nevertheless, further studies are required to determine whether aneurysm morphology itself independently influences long‐term functional recovery.

Unlike SA, the pathology of DA involves a true lumen that communicates with a false lumen through a partial tear of the internal elastic lamina, indicating that the key therapeutic principle is dissection repair (Mizutani et al. 2001). EVT is the preferred primary intervention for DA (Sönmez et al. 2015). EVT can be performed using a deconstructive approach, that is, parent artery occlusion, or a reconstructive approach, including stent‐assisted coil embolization and flow‐diverter stenting (Oh et al. 2022). Parent artery occlusion has been adopted as a treatment option used for selected DA cases, particularly when adequate collateral circulation is confirmed. In the present study, all patients with DA underwent EVT, and parent vessel sacrifice was more frequently performed in the DA group than in the SA group. Although parent artery occlusion carries a theoretical risk of distal ischemia or infarction, the actual risk may depend on individual anatomical and hemodynamic conditions, including collateral circulation and vascular dominance (Foster et al. 2018). Importantly, the decision to perform parent vessel sacrifice is not random but is based on careful evaluation by the treating physicians, which may introduce treatment selection bias. Therefore, the comparable rates of ischemic complications between DA and SA groups should be interpreted cautiously. Rather than indicating that parent vessel sacrifice is universally safe, our findings suggest that this strategy may be feasible in appropriately selected DA patients with favorable collateral circulation.

### 4.1 Limitations

This study has several limitations. First, the retrospective design limits the interpretation of outcomes. Second, although we consecutively included patients with posterior circulation aneurysms from multiple centers, the overall low incidence of posterior circulation aneurysms resulted in insufficient sample sizes for certain aneurysm locations. Third, only patients who underwent surgical or EVT were included. Because some ruptured DA may be managed conservatively, our findings may not represent the entire spectrum of DA. Selection bias due to treatment indication cannot be completely excluded. Finally, the lack of imaging follow‐up may have affected the assessment of treatment efficacy.

## 5 Conclusion

This study is the first to systematically compare clinical outcomes between posterior circulation SA and DA. DA was associated with a trend toward better long‐term functional outcomes compared with SA, although this association was attenuated after adjustment and should be interpreted cautiously. Parent vessel sacrifice was more frequently performed in DA patients, but no significant difference in ischemic complications was observed between the two groups. These findings provide additional clinical evidence for understanding treatment patterns and outcomes of posterior circulation aneurysm subtypes.

## Author Contributions

J. L., G. S., G. L., and X. G. designed the study, collected clinical data, and drafted the initial manuscript. L. L., B. W., J. L., L. F., and Y. T. contributed to data organization, statistical analysis, and visualization of results. J. L., G. S., L. F., X. G., and Y. T. also participated in literature interpretation and manuscript refinement. X. Y., H. L., and X. Z. supervised the overall research process, provided critical revisions for intellectual content, and approved the final version of the manuscript. All authors reviewed and agreed on the final submission.

## Ethics Statement

This study was approved by the Ethics Committee of Tianjin Medical University General Hospital (IRB2022‐YX‐175‐01).

## Funding

This work was supported by the Natural Science Foundation of Tianjin, China (Grant No. 20JCZDJC00300), and the Tianjin Medical University General Hospital Clinical Research Program (Grant No. 22ZYYLCCG07).

## Conflicts of Interest

The authors declare no conflicts of interest.

## Supporting information




**Supplementary Material**: brb371668‐sup‐0001‐SuppMat.docx

## Data Availability

The data supporting the findings of this study are available from the corresponding author upon reasonable request.
