# Supplementary material for: Posterior Circulation Aneurysmal Subarachnoid Hemorrhage: A Comparison Between Saccular and Dissecting Aneurysms
Source: Brain Behav. 2026 Sep 7;16(9):e71668. doi: 10.1002/brb3.71668 (PMC13550871; doi:10.1002/brb3.71668)
Supplement: Supplementary file 1 [file BRB3-16-e71668-s001.docx]

Table S1. Comparison of baseline characteristics between participants lost to follow-up and those who completed follow-up.

|  | Follow-up  (n=332) | Lost-to-Follow-up  (n=74) | P Value |
| --- | --- | --- | --- |
| Age, n (%) |  |  |  |
| <65 | 205 (61.7) | 50 (67.6) | 0.349 |
| ≥65 | 127 (38.3) | 24 (32.4) |  |
| Sex, n (%) |  |  | 0.056 |
| Male | 126 (38.0) | 37 (50.0) |  |
| Female | 206 (62.0) | 37 (50.0) |  |
| Smoking, n (%) | 54 (16.3) | 15 (20.3) | 0.407 |
| Drinking, n (%) | 39 (11.7) | 9 (12.2) | 0.920 |
| Hypertension, n (%) | 222 (66.9) | 42 (56.8) | 0.099 |
| Diabetes, n (%) | 40 (12.0) | 6 (8.1) | 0.334 |
| Previous stroke, n (%) | 48 (14.5) | 11 (14.9) | 0.928 |
| Presence of ICH or IVH, n (%) | 80 (24.1) | 20 (27.0) | 0.597 |
| HH grade, n (%) |  |  | 0.705 |
| I-III | 272 (81.9) | 62 (83.8) |  |
| IV-V | 60 (18.1) | 12 (16.2) |  |
| WFNS grade, n (%) |  |  | 0.886 |
| I-III | 235 (70.8) | 53 (71.6) |  |
| IV-V | 97 (29.2) | 21 (28.4) |  |
| Location of responsible aneurysm, n (%) |  |  | 0.283 |
| BA | 108 (32.5) | 23 (31.1) |  |
| SCA | 13 (3.9) | 3 (4.1) |  |
| PICA | 67 (20.2) | 21 (28.4) |  |
| VA | 74 (22.3) | 12 (16.2) |  |
| Vertebrobasilar junction | 6 (1.8) | 0 |  |
| PCA | 53 (16.0) | 15 (20.3) |  |
| AICA | 11 (3.3) | 0 |  |
| Multiple aneurysms, n (%) | 73 (22.0) | 17 (23.0) | 0.854 |
| Morphology, n (%) |  |  | 0.813 |
| SA | 256 (77.1) | 58 (78.4) |  |
| DA | 76 (22.9) | 16 (21.6) |  |
| Treatment, n (%) |  |  | 0.927 |
| MST | 28 (8.4) | 6 (8.1) |  |
| EVT | 304 (91.6) | 68 (91.9) |  |

Figure S1. Sensitivity analysis assessing the impact of loss to follow-up on 2-year functional outcomes.
